# Synergistic attraction of Western black-legged ticks, *Ixodes pacificus*, to CO_2_ and odorant emissions from deer-associated microbes

**DOI:** 10.1098/rsos.230084

**Published:** 2023-05-17

**Authors:** Justin Long, Keiran Maskell, Regine Gries, Saif Nayani, Claire Gooding, Gerhard Gries

**Affiliations:** Department of Biological Sciences, Simon Fraser University, Burnaby, British Columbia, Canada V5A 1S6

**Keywords:** *Ixodes scapularis*, *Ixodes pacificus*, *Odocoileus virginianus*, sebaceous gland secretions, microbes, semiochemicals

## Abstract

Foraging ticks reportedly exploit diverse cues to locate their hosts. Here, we tested the hypothesis that host-seeking Western black-legged ticks, *Ixodes pacificus*, and black-legged ticks, *I. scapularis*, respond to microbes dwelling in sebaceous gland secretions of white-tailed deer, *Odocoileus virginianus*, the ticks' preferred host. Using sterile wet cotton swabs, microbes were collected from the pelage of a sedated deer near forehead, preorbital, tarsal, metatarsal and interdigital glands. Swabs were plated on agar, and isolated microbes were identified by 16S rRNA amplicon sequencing. Of 31 microbial isolates tested in still-air olfactometers, 10 microbes induced positive arrestment responses by ticks, whereas 10 others were deterrent. Of the 10 microbes prompting arrestment by ticks, four microbes—including *Bacillus aryabhattai* (isolates A4)—also attracted ticks in moving-air Y-tube olfactometers. All four of these microbes emitted carbon dioxide and ammonia as well as volatile blends with overlapping blend constituents. The headspace volatile extract (HVE) of *B. aryabhattai* (HVE-A4) synergistically enhanced the attraction of *I*. *pacificus* to CO_2_. A synthetic blend of HVE-A4 headspace volatiles in combination with CO_2_ synergistically attracted more ticks than CO_2_ alone. Future research should aim to develop a least complex host volatile blend that is attractive to diverse tick taxa.

## 1. Introduction

Ticks are obligate, temporary (intermittent) ectoparasitic blood feeders on vertebrates [1] and vector microbial agents of more animal diseases than any other hematophagous arthropod [2]. To meet their nutritional needs, ticks must be able to locate and detect hosts, and to recognize suitable body regions of their hosts for feeding [3,4]. To locate vertebrate hosts, hard ticks use two strategies [2]. Ambush strategists climb and rest on vegetation such as twigs or blades of grass, where they sense vertebrate host cues such as CO_2_ [5,6], odorants [7], body heat [8], infrared (IR) radiation [9] and vibrations [10]. Ticks then cling onto passing hosts, seek a suitable feeding site and attach. Host-hunting strategists, in turn, actively seek and crawl towards vertebrate hosts, exhibiting taxis towards host-derived cues. Tick species may use one or both strategies, and often alter them, life stage-dependently [2].

During host-foraging, ticks heavily rely on olfaction [11]. The chemoreceptors of ticks, clustered on the Haller's organs of their foreleg tarsi, detect a wide range of semiochemicals (message bearing chemicals) [11] and inform appetence behaviour such as questing and movement towards an odour source [12]. They elicit foraging behaviour in response to odorants of host dermal pelage [13], breath (e.g. CO_2_, 1-octen-3-ol) [5,6], gland secretions [14] and urine [6,7]. Sebaceous gland secretions of host vertebrates provide microhabitats for many microbes [15] which, in turn, produce odorants attractive to ticks [16]. Some rabbit and bovine dermal odorants have already been identified [17–19] but many more remain unknown [1].

The western black-legged tick, *Ixodes pacificus,* and the black-legged tick (deer tick), *I. scapularis* (both hard ticks in the family Ixodidae)*,* are native to North America [20]. Larvae and nymphs of both species parasitize small reptiles and rodents [21,22], whereas adult ticks prefer deer, *Odocoileus* sp*.*, as hosts [21–23] but also feed on other large mammals such as bears, *Ursus americanus* [24]. Both tick species vector the bacterium *Borrelia burgdorferi* which causes Lyme disease in infected individuals [25], affecting more than 300 000 North Americans each year [26].

Deer hosts of *I. pacificus* and *I. scapularis* possess several sebaceous glands such as the tarsal, metatarsal, interdigital, preorbital and forehead gland [27]. *Ixodes* ticks are attracted to objects rubbed with deer pelage from such locations [7], suggesting that ticks sense these gland secretions and/or the odorants stemming from microbial metabolism of these secretions. It follows that ticks may also be attracted to metatarsal and interdigital gland secretions of deer, but this has not yet been tested.

Female *Ixodes* ticks are attracted to butyric acid [28], a well-known bacterial metabolite [29]. Bacteria produce diverse metabolites that may serve as host semiochemical cues for foraging ticks [30–34] (electronic supplementary material, table S1). Many bacterial metabolites, including acids, fatty acid derivatives, aldehydes, and sulfur- and nitrogen-containing compounds [32] have low molecular weight (less than 300 Da) and high vapour pressure (0.01 kPa at 20°C), and thus readily diffuse [32], becoming informative foraging cues to hematophagous ticks and insects. The occurrence of overlap between microbial volatile profiles and known tick attractants [18,35–37] (electronic supplementary material, table S1) suggests that ticks may respond to microbial metabolites during host foraging. Attraction of ticks to gases like carbon dioxide (CO_2_) and ammonia (NH_3_) [36], which are indicative of microbial amino acid metabolism [31], further suggests that host-foraging behaviour by ticks may, in part, be microbe-mediated. Moreover, the distinctively different physico-chemical properties of microbe-emitted gases (e.g. CO_2_, NH_3_) and volatile metabolites may prompt synergistic attraction of ticks to vertebrate hosts, which has not yet been demonstrated. Synergism would be evident, if the interaction between microbial semiochemicals and gases were to produce a combined effect on the attraction of ticks greater than the sum of their separate effects. Mosquitoes have unique CO_2_ and semiochemical receptors that guide them during various stages of host-foraging behaviour [38–40]. Conceivably then, neuronal interactions between gas and odorant receptors may also inform host-foraging by ticks.

Here, we tested the hypotheses (H) that (H1) *I. scapularis* and *I. pacificus* are arrested by, or attracted to, microbes isolated from white-tailed deer, and (H2) attraction of these ticks is mediated synergistically by microbial semiochemicals and gases.

## 2. Material and methods

### 2.1. Collection and storage of microbes

**Figure 1.**
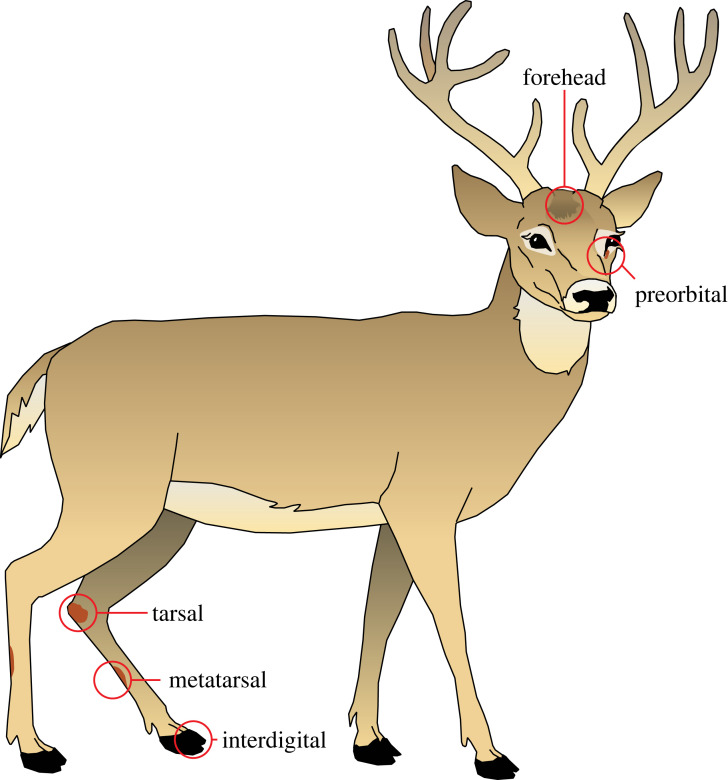
Graphical illustration depicting the locations of forehead, preorbital, tarsal, metatarsal and interdigital excretory glands of white-tailed deer from which swabs were taken for microbe identifications.

Microbes were collected from a male white-tailed deer at a wildlife park in Kamloops, British Columbia (BC) (50°39′14.41″ N 120°4′49.69″ W). After the deer was anaesthetized, separate swabs were taken with a dampened sterile Q-tip cotton swab (Uline Canada, Milton, ON L9T 8L1, CA) from the forehead, preorbital, tarsal, metatarsal, and interdigital glands of the deer (figure 1). An additional single swab was taken from all of these glands. Q-tips were then immediately streaked on separate plates of Mueller Hinton agar (MHA) and incubated 24 h at 32°C. MHA was prepared from beef extract powder (2.0 g), casein acid hydrolysate (17.5 g), soluble starch (1.5 g) (all ingredients: Sigma-Aldrich, Merck KGaA, Darmstadt, DE) and agar bacteriological powder (17.0 g) (ACP Chemicals, Montreal, QC H1R 1A5, CA) in distilled water (1 L) and then autoclaved 45 min at 121°C. Visually distinct microbes (colonies of different colour and/or shape) were isolated by re-plating them in a biosafety cabinet (BSC; NUAIRE Biological Safety Cabinets, Class II type A2), using aseptic techniques. All isolates were assigned a letter code indicating their origin (F, forehead; M, metatarsal; T, tarsal; I, interdigital; E, preorbital; A, all gland regions combined) and a number. A stock plate of each unique microbe was re-plated once a month and stored at 4°C. For long-term storage, microbe stock-samples were kept at –80°C in a solution of glycerol, distilled water and microbe culture (1 : 1 : 2).

### 2.2. Sources and maintenance of ticks

Adult females of *I. pacificus* and *I. scapularis* were obtained from BEI resources (The Biodefense and Emerging Infections Research Resources Repository; managed by the American Type Culture Collection (ATCC), Manassas, VA 20110-2209, USA), and adult female *I. scapularis* also from the State University of Oklahoma. Ticks were housed, separated by species, in groups of 10–12 individuals in 20 ml scintillation vials fitted with a lid with a mesh-covered hole (1 cm) to facilitate ventilation and with thin strips of paper towel as a walk-on substrate. Vials for *I. pacificus* and for *I. scapularis* were kept in two separate desiccators above a saturated K_2_SO_4_ solution that afforded a relative humidity of *ca.* 85%. The desiccators were placed in two plexiglass boxes (34 × 50 × 33 cm high) maintained at a temperature of 20–25°C and exposed to a 12L : 12D photoperiod. While at SFU, ticks were not fed and not re-tested in the same experiment.

### 2.3. H1: ticks are arrested by, or attracted to, microbes isolated from white-tailed deer

#### 2.3.1. Identification of microbes

To prepare microbes for identification, we incubated (24 h at 32°C) visually distinct microbes (see above) on MHA and then circled with a marker the most isolated single colony on the bottom of the plate which was sent to Genewiz (Seattle, WA 98109, USA) for identification. Genewiz amplified the 16S rRNA gene through polymerase chain reaction (PCR) and determined the 16S rRNA gene sequence. Although the 16S rRNA gene is highly conserved among bacterial species, it contains species-specific hypervariable regions which can be used to identify an unknown bacterial species [41]. Results provided by Genewiz consisted of a FASTA sequence which we analysed using the NIH BLAST search tool for regions of similarity between the 16S rRNA gene sequence of a microbial isolate (typically approx. 1550 base pairs (bps) long) and all similar sequences (accession length of most reads: 1450–1550 bps) in the database, eventually producing a table that listed the most likely candidates for the unknown microbe.

#### 2.3.2. Arrestment responses of ticks by microbes in still-air olfactometers

**Figure 2.**
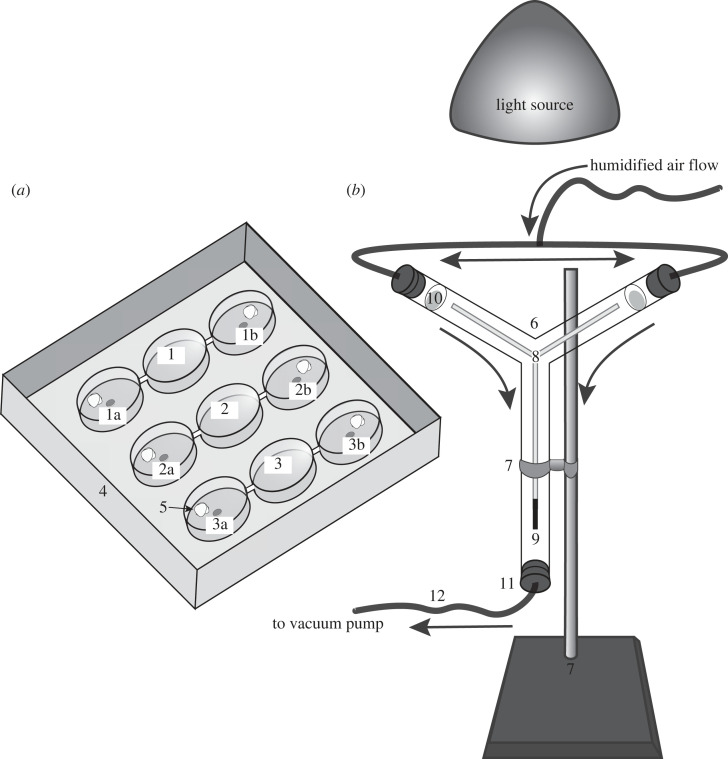
Graphical illustrations of (*a*) still-air 3-chamber olfactometers (1–3) housed in a plexiglass box (4; 33.5 × 49.5 × 9.1 cm high) and (*b*) a moving-air Y-tube olfactometer mounted within a cloth-covered scaffold (57 × 36 × 123 cm; not shown) to standardize visual cues. The lateral chambers (1a, 1b; 2a, 2b; 3a, 3b; each 9.0 × 3.1 cm) of each still-air olfactometer received a 1 cm disc of agar inoculated with a test microbe, or not (control), and a moist sterile cotton ball (5) to elevate the relative humidity. For each bioassay replicate, two ticks were placed in the central chamber of an olfactometer, and after 24 h in darkness their position in the olfactometer was scored (see methods for detail). The Pyrex glass Y-tube olfactometer (6; diameter: 2.0 cm; length of main stem and side arms: 19.5 cm and 11.5 cm, respectively) was held by a clamp (7) and fitted with Y-shaped bamboo skewers (8) as a walk-on substrate for the ticks, with a metal wire (9)—serving as a weight—to prevent physical contact of skewers with the olfactometer. For each replicate, a disc of agar inoculated with a bacterium, or not (control), was placed on sterile filter paper (10) at the orifice of side arms. To initiate a bioassay, a tick was placed on the bottom of the bamboo skewers, and a rubber stopper (11)—connected to a PVC pipe (12) and a vacuum pump—was inserted into the stem of the olfactometer, resulting in a flow (0.2 l·min^−1^) of humidified air (40–70%) through the olfactometer. Each tick that within 12 min walked > 8.0 cm into a treatment or control side arm as its first choice was considered a responder.

The effect of microbes and their volatile metabolites, respectively, on arrestment responses by ticks was tested in still-air, dual-choice olfactometer experiments 1–31 (*n* = 15 or 30 each). Olfactometers consisted of three Pyrex glass chambers (each 3.5 × 10 cm inner diameter (ID) with removable glass lids, linearly interconnected by glass tubes (each 2.5 × 1 cm ID) [42] (figure 2*a*). For each bioassay, a moist sterile cotton ball was added to each lateral chamber to elevate the relative humidity. The randomly assigned lateral treatment chamber also received a disc (1 cm) of MHA excised from a plate inoculated with a test microbe, whereas the lateral control chamber received a disc (1 cm) of sterile MHA. Across all experiments, all treatment plates prior to the onset of bioassays had consistently been incubated 24 h at 32°C. In preliminary bioassays with both lateral chambers of the olfactometer left unbaited, ticks equally often selected the right and the left chamber of olfactometers.

For each bioassay replicate, two ticks (of unknown age since moulting to adults) were transferred via paintbrush from a scintillation vial to the central chamber of an olfactometer. As determined in preliminary bioassays, using two ticks, instead of one, increased the probability that at least one tick responded to test stimuli. After transfer of ticks, all chambers were sealed and closed with parafilm and lids, and placed into plexiglass boxes fitted with a plexiglass lid (figure 2*a*). During bioassays, boxes were kept at a 60–90% relative humidity, 20–23°C, and in complete darkness to eliminate potential phototaxis of ticks towards asymmetries in lighting, as experienced in bioassays with bed bugs, *Cimex lectularius* [43]. Bioassays were initiated by turning off the lights and were terminated 24 h later by switching lights on. Ticks that had entered a lateral treatment or control chamber were considered responders, whereas those that were present in the central (release) chamber were considered non-responders. Drawing on personal experience that insects may reverse decisions during choice bioassays, we scored data 24 h after bioassay initiation to provide ample time for ticks to finalize their decisions.

For each strain of microbe tested, 15 replicates were run initially, but an additional 15 replicates were run, if a microbe elicited significant arrestment responses by ticks. Two strains of microbes, with 15 replicates each, were tested in parallel every day. To ensure no cross contamination, separate Plexiglas boxes were used to test each strain. Between bioassays, the lids of Plexiglas boxes were removed, and the room was ventilated by leaving the door open for at least 1 h.

#### 2.3.3. Attraction of ticks to microbes in moving-air Y-tube olfactometers

**Figure 3.**
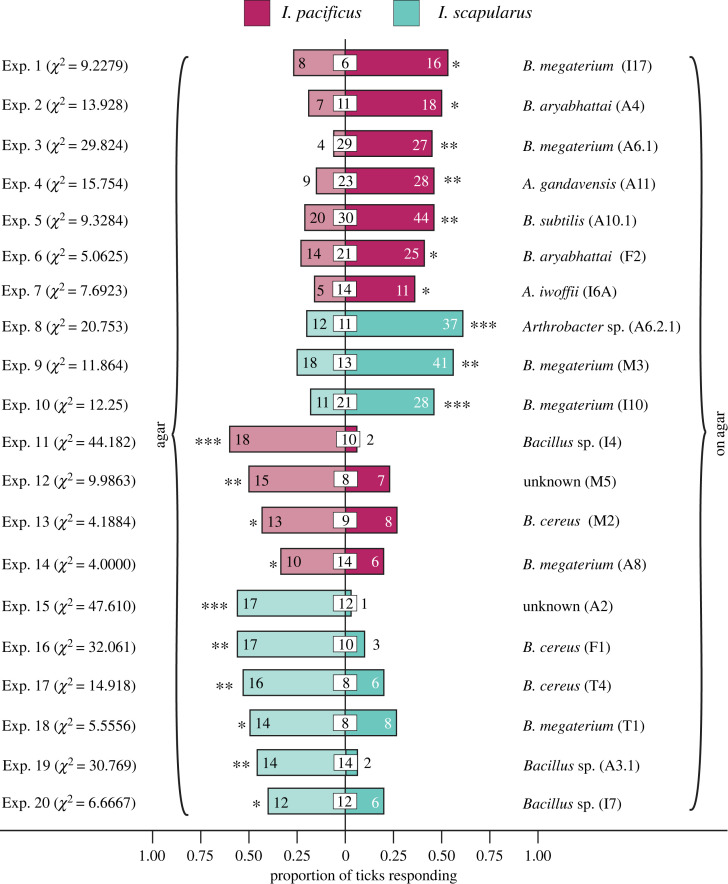
Arrestment of the ticks *Ixodes pacificus* and *I. scapularis* in still-air, three-chamber olfactometer experiments 1–20 (*n* = 15 or 30) (figure 2*a*) in response to microbes collected near excretory glands of white-tailed deer (figure 1). Treatment and control stimuli consisted of microbe-inoculated and sterile agar, respectively. In each replicate, two ticks were tested and the proportion of ticks choosing the lateral chamber with the treatment or with the control stimulus was calculated. An asterisk (*) denotes a significant proportion of ticks arrested in the treatment or the control chamber (*χ*^2^ tests on proportional responses; * = *p* < 0.10, ** = *p* < 0.05, *** = *p* < 0.01).

Attraction of ticks to microbes, shown to be bioactive in still-air olfactometers (see Results; figure 3), was tested in Pyrex glass Y-tube olfactometer experiments 32–39 (*n* = 25 each), modifying a previous design [36]. The olfactometer (diameter: 2.5 cm; length of main stem and side arms: 18.0 cm and 10.5 cm, respectively) was held vertically by a clamp and fitted with Y-shaped bamboo skewers as walk-on substrate for the ticks (figure 2*b*). To prevent physical contact of skewers with the olfactometer, a metal wire—serving as a weight—was attached to the lowermost skewer section. To standardize lighting during behavioural bioassays, all bioassays were run illuminated only by red light (650 nm). To ensure responsiveness of ticks, all bioassays were run at 40–70% relative humidity and 22–25°C.

For each replicate, one-quarter section of an MHA disc (disc diameter: 3.0 cm) covered in a lawn of the test bacterium and one-quarter section of a sterile MHA control disc were placed, by random assignment, on sterile filter paper at the orifice of respective side arms. To initiate flow of odour-laden air, a rubber stopper, connected to a PVC pipe and a vacuum pump (Neptune Dyna-pump, Model 2 Dover, NJ, USA), was inserted into the stem of the olfactometer, drawing humidified air (40–70%) at a rate of 0.2 l·min^−1^ for 2 min through the olfactometer. Then, the stopper was removed, an active tick—as determined by its questing response to exhaled breath—was placed on the bottom of the bamboo skewers, and the olfactometer was re-connected to the vacuum pump. Each tick that proceeded 8.0 cm or more into either a treatment or control side arm as its first choice within 12 min was considered a responder. All other ticks were considered non-responders.

To kill any bacteria that may have been pulled from the treatment disc, the air exiting the olfactometer was also drawn through a gas bubbler (height = 26.5 cm: top width = 4.0 cm: bottom width = 15.0 cm) containing 70% ethanol and then vented out of the bioassay room to prevent ethanol accumulation and thus adverse effects on the ticks' behavioural responses. Prior to testing a new strain of microbe, the door of the bioassay room was opened for 5 min, allowing fresh air to enter.

### 2.4. H2: attraction of ticks is mediated synergistically by microbial semiochemicals and gases

#### 2.4.1. Collection of headspace volatiles from microbes

Headspace volatiles were collected from all strains of microbes that elicited significant behavioural responses by ticks in Y-tube olfactometer experiments. To this end, 12 MHA plates were inoculated with a microbe of interest and incubated for approximately 20 h. These plates, with open lids, were then placed into an aeration chamber (diameter = 19 cm: height = 29.5 cm) connected to a vacuum pump (Neptune Dyna-pump). Charcoal-filtered air was drawn at a flow rate of 1 l·min^−1^ for 20 h through the chamber and through a glass column (6 mm outer diameter × 150 mm) containing 200 mg of manufacturer-preconditioned Porapak-Q adsorbent (50–80 mesh; Waters Associates, Milford, MA, USA), or 200 mg of the adsorbents Carbosieve SIII (60/80 mesh; Supelco, PA, USA) and Tenax TA (35/60 mesh, Chromatographic Specialties, Brockville, ON, CA) (2.7 : 1). Volatiles were desorbed with one rinse of ether (2 ml), which then contained 240 plate-hour-equivalents (240 PHEs; 12 microbe-inoculated ager plates × 20 h of volatile captures) of headspace volatile extract (HVE). Volatile extracts were concentrated to 0.5 ml under a stream of nitrogen, and were kept at 4°C prior to analyses. All glassware was cleaned with Sparkleen (Thermo Fisher Scientific, MA, USA), rinsed with distilled water, and oven-dried at 130°C prior to starting a new aeration.

#### 2.4.2. Analyses of microbe headspace volatiles by gas chromatography-mass spectrometry

Aliquots of microbe HVEs were analysed by gas chromatography-mass spectrometry (GC-MS), using both an Agilent 5977A coupled to an Agilent 7890B GC (Agilent Technologies Inc., Santa Clara, CA, USA), and a Varian Saturn Ion Trap GC-MS coupled to Varian 3800 GC. The Agilent instrument was operated in full-scan electron ionization mode and fitted with an MS DB-5 column (30 mm × 0.25 mm ID, film thickness 0.25 µm; Agilent Technologies), using helium as the carrier gas (35 cm · s^−1^). The oven temperature program was as follows: 40°C (held for 5 min), 10°C · min^−1^ to 280°C (held for 10 min). The injector was operated in split mode (5 : 1 ratio), the injector port was set to 250°C, the transfer line to 280°C, the MS Quadrupole to 150°C and the MS source to 230°C. The Saturn Ion Trap and Manifold were set to 200°C and 80°C, respectively, the GC was fitted with a DB-5 MS GC column (see above), and the temperature program was as follows: 35°C for 10 min, then 10°C per min to 280°C (0 min hold). Compounds were identified by comparing their retention indices [44] and mass spectra with those of authentic standards that were purchased or synthesized (table 1). Each compound was quantified by comparing its area count with that of an external standard (1 or 10 ng of 2-nonanone).

**Table 1.** Compounds (ng) present in a synthetic blend were prepared according to headspace volatiles of *Bacillus aryabhattai* (A4) at a dose of 4.8 plate-hour-equivalents (amount of volatiles released during 1 h from 4.8 plates of agar inoculated with *B. aryabhattai*) in 10 µl of ether. The suppliers of synthetic chemicals (with purities in parenthesis) are listed in the right column.

**chemical**	**amount (ng)**	**supplier (% purity)**
acetic acid	5	Anachemia (99)
thioacetic acid methyl ester	2.5	Esterified from thioacetic acid, Sigma-Aldrich (96)
butanal	1	Sigma-Aldrich (97)
2-butanone	5	EMD (99)
3-methyl butyric acid	5	Sigma-Aldrich (98)
2-methylbutyric acid	5	Sigma-Aldrich (98)
3-methylbutanol	2.5	fisher (>95)
methylthiocyanate	2.5	Sigma-Aldrich (97)
3-methylbutanal	1	Sigma-Aldrich (97)
dimethyltrisulfide	15	Sigma-Aldrich (98)
2-pentanone	5	Sigma-Aldrich (99)
4-methyl-2-pentanone	1.25	Sigma-Aldrich (98)
3-methyl-2-pentanone	1.25	Sigma-Aldrich (98)
2-hexanone	1	Sigma-Aldrich (98)
5-methyl-2-hexanone	2.5	Sigma-Aldrich (99)
2-heptanone	2.5	Sigma-Aldrich (98)
6-methyl-2-heptanone	2.5	Sigma-Aldrich (>95)
8-methyl-2-nonanone^a^	2.5	synthesized in the Gries-laboratory
2,5-dimethylpyrazine	500	Sigma-Aldrich (98)
2-isopropyl-5-methylpyrazine^b^	10	synthesized in the Gries-laboratory

^a^7-Methyl-1-octanol (Toronto Research Chemical) was oxidized to the aldehyde; then, a Grignard reaction with 7-methyloctanal and methylmagnesium bromide afforded 8-methyl-2-nonanol which was oxidized to the ketone.

^b^Synthesized as previously described [45,46].

For thermal desorption and identification of microbe-derived headspace volatiles, we used a 7694 Agilent Headspace Sampler coupled to a Saturn Ion Trap GC-MS (see above) fitted with a DB-5 MS GC column (see above). Microbe headspace volatiles were first captured for 24 h on Carbosieve/Tenax (see above), and then these two adsorbents were placed into a 20 ml vial which was sealed with a crimped cap with a 20 mm OD white silicon septum and heated to 180°C for 5 min. The thermally desorbed volatiles were withdrawn with an automated syringe and subjected to GC-MS analysis (5:1 split ratio; filaments turned on after 10 s), using the following temperature program: 35°C for 10 min, then 10°C per min to 280°C. To quantify volatiles found exclusively in this type of analysis and to integrate these volatiles in a complex synthetic blend (table 1), the ratio between the same volatiles that were desorbed thermally, or by solvent from Carbosieve/Tenax, was calculated and used as a correction factor.

#### 2.4.3. Detection of NH_3_ and CO_2_ released from microbes and watery solutions of ammonium bicarbonate and ammonium hydroxide

To determine the presence of microbe-produced ammonia (NH_3_), we rented a calibrated RAE—MultiRae PGM 6228 meter (Pine Environmental Services LLC, Burnaby, BC, CA) and placed the meter's hose (connected to a Hepa filter at the inlet) approximately 0.5 cm above the MHA plate which had been incubated with a microbe of interest. Within 3 min of sampling, the concentration of NH_3_ was recorded.

To determine the presence of microbe-produced CO_2_, we used a Q-Trek 7575x meter with probe 982. MHA that had been incubated with a microbe of interest was inserted into a 1 l jar through its 4 cm wide neck, and the probe—with the CO_2_ sensors located 10–15 cm away from its tip—was positioned 1 cm above the MHA. Parafilm was stretched over the jar's orifice to separate the environments within and outside the jar. The ambient CO_2_ concentration (*ca.* 400 ppm) outside the jar was subtracted from the in-jar reading which was recorded within 3 min.

To determine NH_3_ dissemination from ammonium bicarbonate (NH_4_HCO_3_), 5 g of NH_4_HCO_3_ were dissolved in sugar water (25 g sugar: 20 ml H_2_O at room temperature), and NH_3_ measurements were taken 1.6 cm above the liquid, using the MultiRae PGM 6228 meter. Similarly, to determine NH_3_ dissemination from ammonium hydroxide (NH_4_OH), a 12.5 µl solution (containing 30% NH_4_OH) was dissolved in water (25 ml), and NH_3_ measurements were taken 1.6 cm above the liquid.

Dissemination of CO_2_ from the watery NH_4_HCO_3_ solution could not be quantified in an open-air setting because the probe could not be brought sufficiently close to the liquid surface (see above). However, CO_2_ dissemination from the NH_4_HCO_3_ solution was confirmed inside a 1 l jar. One minute after placing the solution in the jar and closing it, the CO_2_ concentration had risen to 1200 ppm above ambient.

#### 2.4.4. Attraction of ticks to natural or synthetic microbe volatiles and gases

Experiments 40–51 followed the same general Y-tube olfactometer design as described above and tested responses of ticks to a set of diverse stimuli listed in table 2 and described below. The flow rate for these bioassays was 200 ml· min^−1^ (Exps. 32–39) or 75 ml· min^−1^ (Exps. 40–51). Between replicates, the door of the bioassay room was opened to ensure air exchange.

**Table 2.** Summary of experiments (Exps.) and replicates (*n*) run to test for attraction of the ticks *Ixodes pacificus* and *I. scapularis* in Y-tube olfactometers (figure 2*b*) to (*i*) microbe-derived gases (Exps. 40–42), (*ii*) headspace volatile extract of *Bacillus aryabhattai* (A4) (HVE-A4) (Exp. 43), (*iii*) HVE-A4 *plus* CO_2_ versus CO_2_ (Exps. 44–48), (*iv*) HVE-A4 *plus* CO_2_ versus HVE-A4 (Exp. 49) and (*v*) synthetic HVE-A4 *plus* CO_2_ versus CO_2_ (Exps. 50–51).

exp. # (*n*)	control stimulus	treatment stimulus	tick sp. tested
*effect of microbe gases on tick attraction*			
40 (25)	sugar water (25 g/20 ml)	(NH₄)₂CO₃^a^ (5 g) in sugar water (25 g/20 ml)	*pacificus*
41 (25)	water (25 ml)	NH_4_OH^b^ (12.5 µl) in water (25 ml)	*pacificus*
42 (25)	medical-grade air^c^	CO_2_ (4%) in medical-grade air (75 ml/min)^c^	*pacificus*
*effect of microbe headspace volatile extract on tick attraction*			
43 (25)	ether (10 µl)^e^	HVE-A4 (4.8 PHEs)^d^ in ether (10 µl)^e^	*pacificus*
*interactive effect between microbe headspace volatile extract and CO_2_ on tick attraction*			
44 (25)	ether + CO_2_	HVE-A4 (4.8 PHEs)^g^ in ether + CO_2_	*pacificus*
45 (25)	ether + CO_2_	HVE-A4 (4.8 PHEs)^g^ in ether + CO_2_	*pacificus*
46 (12)	ether + CO_2_	HVE-A4 (0.048 PHEs) in ether + CO_2_	*pacificus*
47 (25)	ether + CO_2_	HVE-A4 (4.8 PHEs) in ether + CO_2_	*scapularis*
48 (25)	ether + CO_2_	HVE-A4 (4.8 PHEs) in ether + CO_2_	*scapularis*
49 (25)	HVE-A4 (4.8 PHEs) in ether + air	HVE-A4 (4.8 PHEs) in ether + CO_2_ in air	*pacificus*
*interactive effect between synthetic microbe volatiles and CO_2_ on tick attraction*			
50 (25)	ether + CO_2_	synthetic^f^ HVE-A4 (4.8 PHEs) in ether + CO_2_	*pacificus*
51 (25)	ether + CO_2_	synthetic^f^ HVE-A4 (48 PHEs) in ether + CO_2_	*pacificus*

^a^Ammonium bicarbonate emitting CO_2_ (quantifiable in closed jar but not in open-air setting; see methods) and NH_3_ (4–6 ppm 1.6 cm above the liquid).

^b^Ammonium hydroxide emitting NH_3_ (4–6 ppm 1.6 cm above the liquid; see methods).

^c^In all experiments that involved CO_2_, CO_2_ (4%) was delivered in medical grade air at a flowrate of 75 ml · min^−1^.

^d^Headspace volatile extract of *Bacillus aryabhattai* (A4) (HVE-A4) tested at 4.8 plate-hour-equivalents (4.8 PHEs = amount of volatiles released during 1 h from 4.8 plates of agar inoculated with *B. aryabhattai*).

^e^The test volume of ether in all experiments was 10 µl.

^f^For blend composition see table 1.

^g^Two separate extracts were tested in experiments 44 and 45, and in experiments 47 and 48.

#### 2.4.5. Effect of microbe gases on tick attraction

To test for the attraction of *I. pacificus* to NH_3_ and CO_2_ (table 2, Exp. 40), two stimuli were prepared. The treatment stimulus consisted of ammonium bicarbonate (NH_4_HCO_3_) (5 g) dissolved in sugar water (25 g sugar: 20 ml H_2_O) disseminating both NH_3_ (4–6 ppm 1.6 cm above the liquid) and CO_2_ (not quantifiable in open-air setting, see above), and the control stimulus consisted of sugar water only (25 g sugar: 20 ml H_2_O) disseminating neither gas. For each replicate, a vial lid containing 500 µl of the treatment or the control stimulus was placed in a randomly assigned side arm of the olfactometer and kept in place by a metal mesh.

To test for attraction of *I. pacificus* to NH_3_ only [see 36] (table 2, Exp. 41), the treatment stimulus consisted of ammonium hydroxide (NH_4_OH; 30% in water) (12.5 µl) further diluted in water (25 ml) disseminating NH_3_ (6–7 ppm 1.6 cm above the liquid), whereas the control stimulus consisted of water only (25 ml). For each replicate, a sterile piece of filter paper (see above) was treated with 25 µl of the treatment or the control stimulus, and was placed—by random assignment—at the end of the left or right olfactometer side arm.

To test the effect of CO_2_ on *I. pacificus* attraction [see 36] (table 2, Exp. 42), the treatment stimulus consisted of 4% CO_2_ in medical grade air (custom-formulated by Praxair/Linde Canada Inc., Surrey, BC V4N 5L8, CA) and the control stimulus consisted of medical grade air (Linde Canada). For each replicate, tubing from either cylinder of air was funneled through a rubber stopper, and the stopper was inserted into a side arm.

#### 2.4.6. Effect of microbe headspace volatile extract on tick attraction

**Figure 4.**
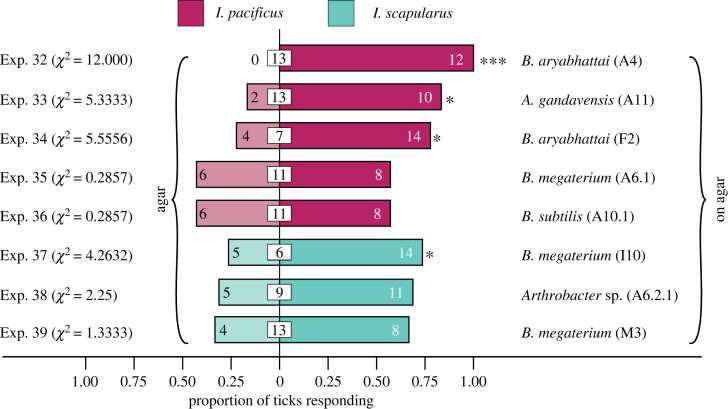
Attraction of the ticks *Ixodes pacificus* and *I. scapularis* in Y-tube binary choice olfactometer bioassays (figure 2*b*) in response to microbes collected near excretory glands of white-tailed deer (figure 1). Treatment and control stimuli consisted of microbe-inoculated and sterile agar, respectively (*χ*^2^ tests on absolute numbers; * = *p* < 0.10, *** = *p* < 0.01).

To test the effect of HVE of bioactive microbe A4 (HVE-A4), rather than A4 itself (figure 4), on *I. pacificus* attraction (table 2, Exp. 43), the treatment stimulus consisted of HVE-A4 extract (4.8 PHEs in 10 µl ether) and the control stimulus was an equal volume of ether. For each replicate, a sterile piece of filter paper (see above) was treated with 10 µl of the treatment or the control stimulus, and was placed in the lower section of the left or right olfactometer side arm. A dose of 4.8 PHEs was chosen for bioassays to ensure that potential microbe-derived trace semiochemicals (which can be lost during desorption from volatile traps) were present at quantities sufficiently high to elicit behavioural responses by ticks.

#### 2.4.7. Interactive effect between microbe headspace volatile extract and CO_2_ on tick attraction

To test whether HVE-A4 enhances the attractiveness of CO_2_ to ticks (table 2, Exps. 44–48), CO_2_ (4%) enriched medical air was introduced into both the treatment and the control arm (net flow rate: 75 ml · min^−1^). The treatment arm of the olfactometer received a piece of filter paper (see above) treated with HVE-A4 extract in ether (10 µl) at one of two doses for *I. pacificus* (4.8, 0.048 PHEs; Exps. 44–46) and one dose for *I. scapularis* (4.8 PHEs; Exps. 47–48), whereas the filter paper in the control arm received 10 µl of ether. To confirm behavioural responses of ticks to HVE-A4, two separate HVE-A4 extracts at a dose of 4.8 PHEs were tested with *I. pacificus* (Exps. 44–45) and with *I. scapularis* (Exps. 47–48).

To test whether CO_2_ enhances the effect of HVE-A4 (table 2, Exp. 49) on the attraction of *I. pacificus*, both the treatment and the control arm of the olfactometer received a piece of filter paper (see above) treated with HVE-A4 at 4.8 PHEs. Medical air enriched with 4% CO_2_ was introduced into the treatment arm, whereas plain medical air was introduced into the control arm.

#### 2.4.8. Interactive effect between synthetic microbe volatiles and CO_2_ on tick attraction

To test for attraction of *I. pacificus* to a 20-component synthetic blend of HVE-A4 volatiles (table 2, Exps. 50–51), the synthetic blend (table 1) was presented at doses equivalent to those of HVE-A4 at 4.8 PHEs, and at 48 PHEs, with the solvent ether serving as the corresponding control stimulus. Medical air enriched with 4% CO_2_ was introduced into both the treatment and the control arm.

### 2.5. Statistical analyses

Data of experiments 1–51 were analysed in R-studio [47], using an *χ*^2^ test to compare proportions, or numbers, of ticks choosing treatment or control stimuli. In still-air three-chamber olfactometer experiments 1–31 (which tested two ticks in each replicate), the proportions of ticks choosing the lateral chamber with the treatment, or the control stimulus were calculated and analysed. If, after 15 replicates in any of experiments 1–31, a *p*-value of < 0.10 was calculated, 15 additional replicates were completed to avoid statistical error type II. If a microbial isolate in 30 replicates induced preferential arrestment of ticks (*p* < 0.10) in the microbe-baited treatment chamber, that microbial isolate was further tested in moving-air Y-tube olfactometer experiments 32–39 (*n* = 25 each), setting the α-value to 0.05. The same α-value (0.05) was used in experiments 40–51. In experiments 1–31, the threshold for statistical significance was set higher (*p* < 0.10) to ensure that potentially bioactive microbes were not erroneously eliminated from further rigorous testing in Y-tube olfactometers.

## 3. Results

### 3.1. H1: ticks are arrested by, or attracted to, microbes isolated from white-tailed deer

#### 3.1.1. Identification of microbes

Of 31 microbial isolates collected near excretory glands of deer, eight species in seven genera were identified by sequencing (table 3). Of these eight species, *Bacillus megaterium* accounted for 11 unique isolates. Three of these *B. megaterium* isolates (I17, T1, T9.1) were identified as ATCC 14581, even though they differed morphologically. Both F2 and A4 were identified as *Priestia aryabhattai* strain B8W22. From the NIH BLAST database, seven microbes (A1, A3.1, A6.2.1, I4, I7, I9 and T6) could be identified only to the genus level. The 16S rRNA gene sequences for microbes A2 and M5 had no matches in the NIH BLAST database and remain unknown.

**Table 3.** List of microbes collected by cotton swabs around gland secretions from a male white-tailed deer and identified by 16S amplicon sequencing, with accession numbers of the sequences obtained. Isolates were assigned a letter code indicating their origin (A, all gland regions combined; I, interdigital; F, forehead; M, metatarsal; T, tarsal; E, preorbital) and a number.

microbe genus and species	microbe ID	gland(s)	accession numbers
*Bacillus* sp.	A1	all	1533
unknown	A2	all	n.a.
*Bacillus* sp.	A3.1	all	1533
*Bacillus aryabhattai*	A4	all	1533
*Bacillus megaterium*	A6.1	all	1477
*Arthrobacter* sp.	A6.2.1	all	1499
*Staphylococcus warneri*	A7.3	all	1470
*Bacillus megaterium*	A8	all	1495
*Bacillus subtilis*	A10.1	all	1487
*Arthrobacter gandavensis*	A11	all	1445
*Paenarthrobacter nitroguajacolicus*	A12	all	1488
*Bacillus* sp.	I4	interdigital	1544
*Exiguobacterium* sp.	I6A	interdigital	1550
*Bacillus* sp.	I7	interdigital	1509
*Bacillus* sp.	I9	interdigital	1509
*Bacillus megaterium*	I10	interdigital	1477
*Acinetobacter iwofii*	I16A	interdigital	1460
*Bacillus megaterium*	I17	interdigital	1495
*Bacillus cereus*	F1	forehead	1486
*Bacillus aryabhattai*	F2	forehead	1533
*Bacillus megaterium*	M1	metatarsal	1467
*Bacillus cereus*	M2	metatarsal	1486
*Bacillus megaterium*	M3	metatarsal	1477
unknown	M5	metatarsal	n.a.
*Bacillus megaterium*	M9	metatarsal	1501
*Bacillus megaterium*	M10.1	metatarsal	1477
*Bacillus megaterium*	T1	tarsal	1477
*Bacillus cereus*	T4	tarsal	1486
*Bacillus* sp.	T6	tarsal	1434
*Bacillus megaterium*	T9.1	tarsal	1495
*Bacillus megaterium*	E3.2	preorbital	1503

#### 3.1.2. Arrestment of ticks by microbes in still-air olfactometers

Ten of the 31 microbial isolates (*Bacillus* (*Priestia*) *megaterium* (isolates I17, A6.1, M3 and I10)*, Bacillus* (*Priestia*) *aryabhattai* (isolates A4 and F2), *Arthrobacter gandavensis* (A11), *Bacillus subtilis* (A10.1), *Acinetobacter iwofii* (I6A) and *Arthrobacter* sp. (A6.2.1)) prompted arrestment of ticks in microbe-baited olfactometer chambers, indicating positive responses by ticks (figure 3). Conversely, 10 other isolates (*Bacillus* sp. (I4, A3.1 and I7), unknown (M5 and A2), *B. cereus* (M2, F1 and T4) and *B. megaterium* (A8 and T1)) prompted arrestment of ticks in unbaited control chambers (figure 3), indicating microbe aversion behaviour by ticks. Additional isolates had no behaviour-modifying effect on ticks (electronic supplementary material, table S2; Exps. 21–31). There was no apparent pattern indicating that the gland location from which microbes originated had a positive or aversive effect on responses of ticks.

#### 3.1.3. Attraction of ticks to host microbes in Y-tube olfactometers

All microbial strains, except *Acinetobacter iwofii* (I16A) and *Bacillus megaterium* (I17) which were erroneously omitted, that elicited positive arrestment responses in still-air olfactometer experiments (figure 3) were further tested in Y-tube olfactometer experiments. Four of these eight strains—*Bacillus aryabhattai* (A4), *Arthrobacter gandavensis* (A11), *Bacillus aryabhattai* (F2) and *Bacillus megaterium* (I10)—also attracted ticks in moving-air Y-tube olfactometer experiments (figure 4). The remaining four strains induced neither attraction nor repellent responses by ticks.

### 3.2. H2: attraction of ticks is mediated synergistically by microbial semiochemicals and gases

#### 3.2.1. Analyses of headspace volatiles and gases from microbes attractive to ticks

In Porapak Q HVEs of the four microbial isolates (*Bacillus aryabhattai* (A4), *Arthrobacter gandavensis* (A11), *Bacillus aryabhattai* (F2), *Bacillus megaterium* (I10)) shown to be attractive to ticks in Y-tube olfactometer experiments (figure 4), 15 volatiles were identified including three acids, six heterocyclic aromatic pyrazines and six ketones (table 4). Several compounds were present in HVEs of all isolates, whereas other compounds were unique to one isolate, such as isobutyric acid to *Bacillus megaterium* (I10)*,* 2-ethyl-3,6-dimethylpyrazine and 2-ethyl-3,5-dimethylpyrazine to *Bacillus aryabhattai* (A4), 5-methyl-2-heptanone to *Bacillus megaterium* (I10) and phenyl-2-propanone to *Arthrobacter gandavensis* (A11) (table 4). All four isolates emitted CO_2_ and NH_3_ (table 4), with *Arthrobacter gandavensis* (A11) emitting the highest concentration (parts per million) of both gases.

**Table 4.** List of volatiles identified in Porapak Q headspace volatiles extracts of the four microbial isolates (*Bacillus aryabhattai* (A4, F2), *Arthrobacter gandavensis* (A11), *Bacillus megaterium* (I10)) that attracted ticks in Y-tube olfactometer experiments (figure 2*b*; figure 4). The per cent (%) relative abundance of each volatile in its respective blend is reported, as is the volume (parts per million) of two gases, carbon dioxide (CO_2_) and ammonium (NH_3_), produced by each isolate.

volatiles and gases	% relative abundance in blend			
	A4	A11	I10	F2
isobutyric acid	0	0	0.73	0
3-methylbutyric acid	0.43	0	3.68	0.10
2-methylbutyric acid	0.38	0	3.16	0.19
2,5-dimethylpyrazine	91.88	0	80.51	91.83
trimethylpyrazine	4.72	0	7.96	5.41
5-isopropyl-2-methylpyrazine	1.41	0	1.66	1.84
2-ethyl-3,6-dimethylpyrazine	0.16	0	0	0
2-ethyl-3,5-dimethylpyrazine	0.41	0	0	0
5-methyl-2-hexanone	0.29	0	0	0.28
6-methyl-2-heptanone	0	0	0.53	0.35
5-methyl-2-heptanone	0	0	1.45	0
phenyl-2-propanone	0	100	0	0
8-methyl-2-nonanone	0.12	0	0	0
7-methyl-2-nonanone	0.15	0	0	0
ammonium (NH_3_)	14.67 ppm	17.33 ppm	13.0 ppm	12.67 ppm
carbon dioxide (CO_2_)	650.0 ppm	800.0 ppm	700.0 ppm	655.0 ppm

The Tenax/Carbosieve HVE of *Bacillus aryabhattai* (A4) contained a blend of volatiles similar to that of Porapak Q HVE but also contained some additional volatiles such as acetic acid, thioacetic acid methyl ester, 2-butanone and 2-pentanone (electronic supplementary material, table S3).

Most of the thermally desorbed headspace volatiles were also detected in solvent extracts of Porapak Q and Tenax/Carbosieve (electronic supplementary material, table S3), but four chemicals were found exclusively in this type of analysis: butanal, 3-methylbutanal, 3-methylbutanol and methylthiocyanate.

#### 3.2.2. Attraction of ticks in Y-tube olfactometers to microbe headspace volatile extracts and to synthetic microbial semiochemicals and gases

**Figure 5.**
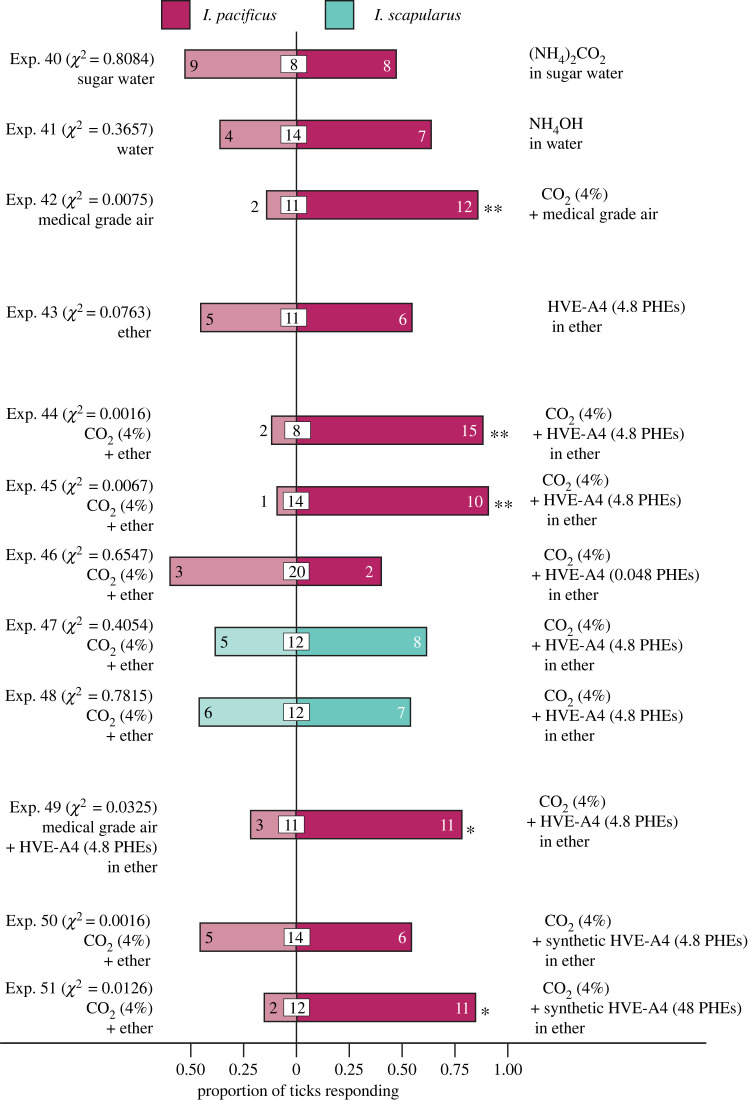
Responses of *Ixodes pacificus* and *I. scapularis* in Y-tube olfactometers (figure 2*b*) to (i) microbe-derived gases (Exps. 40–42), (ii) Tenax/Carbosieve headspace volatile extract of *Bacillus aryabhattai* (A4) (HVE-A4) (Exp. 43), (iii) HVE-A4 *plus* CO_2_ (4% in medical air) versus CO_2_ (*I. pacificus*: Exps. 44–46; *I. scapularis*: 47–48), (*iv*) HVE-A4 *plus* CO_2_ versus HVE-A4 (Exp. 49) and (*v*) synthetic HVE-A4 plus CO_2_ versus CO_2_ (Exps. 50–51) (*χ*^2^ tests on absolute numbers; * = *p* < 0.05, ** = *p* < 0.01). Note: (1) synthetic blend composition reported in table 1; (2) all volatile blends and corresponding solvent controls were applied in 10 µl aliquots; (3) 4.8 PHE = amount of volatiles released during 1 h from 4.8 plates of agar inoculated with *B. aryabhattai*.

The effect of gases on tick attraction was dependent on both the gas and its delivery system. NH_3_ and CO_2_ emanating together from ammonium bicarbonate in water did not attract *I. pacificus* (figure 5, Exp. 40), nor did NH_3_ emanating from ammonium hydroxide in water (figure 5, Exp. 41). By contrast, CO_2_-enriched (4%) medical air significantly attracted *I. pacificus* (figure 5, Exp. 42).

HVE of bioactive microbe A4 (HVE-A4; 4.8 PHEs) on its own failed to attract *I. pacificus* (figure 4, Exp. 43) but HVE-A4 (4.8 PHEs in each of two separate extracts) in combination with CO_2_ attracted significantly more *I. pacificus,* but not *I. scapularis,* than CO_2_ alone (figure 5, Exps. 44–45 and 47–48). A lower dose of HVE-A4 (0.048 PHEs) in combination with CO_2_ was not more attractive to *I. pacificus* than CO_2_ (figure 5, Exp. 46), indicating that the synergistic effect of HVE-A4 is dose-dependent.

The synergistic interaction between HVE-A4 (4.8 PHEs) and CO_2_ was also evident in that this binary combination attracted significantly more *I. pacificus* than HVE-A4 alone (figure 4, Exp. 49). Similarly, synthetic HVE-A4 at 48 PHEs, but not at 4.8 PHEs, *plus* CO_2_ attracted significantly more *I. pacificus* than CO_2_ alone (figure 5, Exps. 50–51), substantiating the synergistic effect of volatiles and CO_2_ produced by *Bacillus aryabhattai* (A4) on the attraction of *I. pacificus*.

In experiments 44, 45, 49 and 51, the binary combination of microbial semiochemicals and CO_2_ attracted 7.5, 10.0, 3.7 and 5.5 times more ticks, respectively, than either the semiochemicals or CO_2_ on their own. As microbial semiochemicals and CO_2_ produced a combined effect on the attraction of ticks greater than the sum of their separate effects, the interaction is synergistic rather than additive.

## 4. Discussion

Our data support the hypotheses (i) that *I. pacificus* and *I. scapularis* are arrested by, and attracted to, skin-dwelling microbes of white-tailed deer, and (ii) that attraction of *I. pacificus* is mediated by specific microbe-derived semiochemicals and CO_2_.

Inspired by findings that the human skin microbiome emits volatiles that affect host-foraging behaviour by mosquitoes [48–50], we investigated whether skin-dwelling microbes of deer affect foraging decisions by ticks (*I. pacificus* and *I. scapularis*) that commonly seek deer as hosts. Our research was further motivated by reports that compounds attractive to ticks are also known microbial metabolites (electronic supplementary material, table S1) but that definitive proof for tick attraction to microbes and their metabolites was still missing.

To collect microbes, we took cotton swabs from pelage areas of deer closely surrounding sebaceous glands, anticipating that the nutrient-rich gland secretions would support a thriving microbial flora [51]. Through careful and repetitive re-plating of deer pelage microbes, 31 morphologically distinct colonies were isolated and identified by 16S amplicon sequencing. Although morphologically distinct, multiple isolates were identified as the same species (table 3). For example, A6.1, A8, I10, I17, M1, M3, M9, M10.1, T1, T9.1 and E3.2 were all identified as *Bacillus megaterium*. However, only one of these isolates, I10, attracted ticks in Y-tube olfactometers (figure 4), indicating that *B. megaterium* in the deer skin microbiome consists of strains with divergent volatile profiles. Similarly, strains of *B. subtilis* isolated from different habitats revealed isolate-specific volatile emission patterns [52]. Discounting all the strains of the same microbe species, we still found as many as eight species in seven genera in secretions from five sebaceous glands of deer (table 3).

To assess whether microbial isolates induced behavioural responses by ticks, we first tested isolates in still-air olfactometer experiments. By running 30 replicates concurrently at any time, many isolates could be efficiently screened for their behavioural effects on ticks. Of 31 microbial isolates growing on agar and placed in a lateral chamber of olfactometers (figure 2*a*), 10 isolates induced arrestment responses by ticks, whereas 10 other isolates prompted aversion responses, with ticks residing in unbaited control chambers at the time of experiment termination. Although we did expect to find microbes that induce arrestment by ticks, we did not expect to find microbes, such as *Bacillus* spp. (I4, A3.1, I7) and *B. cereus* (M2, F1, T4), that deter ticks (figure 3). These deterrent microbes, however, may play a role in guiding ticks away from micro-locations of deer skin that are less suitable or preferred for obtaining a blood meal [3,4]. Moreover, not all microbes that comprise the microbiome of prospective vertebrate hosts are necessarily attractive to hematophagous arthropods. Human hosts greatly differ in their attractiveness to host-foraging mosquitoes due, in part, to their contrasting microbiomes and the volatiles they emit [50]. Whether ticks are attracted to, or repelled by, a deer host may depend upon the relative proportions of attractive and deterrent microbes that make up the microbiome.

In all still-air olfactometer experiments, we scored whether ticks had arrested in the microbe-baited chamber or the unbaited control chamber of olfactometers at the time of experiment termination. As we did not observe the ticks' responses during the 24-h experiments, it remained unknown whether ticks were arrested by microbes when they closely approached or whether they were attracted (or repelled) by microbes. To test for attraction of ticks to microbes, we ran moving-air Y-tube olfactometer experiments, where ticks move towards natural or synthetic odour sources. In Y-tube olfactometer experiments, *Bacillus aryabhattai* (A4, F2) and *Arthrobacter gandavensis* (A11) significantly attracted *I. pacificus*, whereas *Bacillus megaterium* (I10) significantly attracted *I. scapularis* (figure 4). Trend-wise, *Arthrobacter* sp. (A6.2.1) and *Bacillus megaterium* (M3) also attracted *I. scapularis* (treatment to control response ratio 11:5 and 8:4, respectively) but the sample size was not sufficiently large to demonstrate a statistically significant effect. To demonstrate attraction, rather than just arrestment, of ticks in response to microbes or synthetic semiochemicals is essential for the development of synthetic trap lures because their efficacy for tick control relies on tick attraction to traps or lethal sources.

To identify the volatiles of the four bioactive isolates that mediated tick attraction (figure 4), we collected their headspace volatiles on Porapak Q adsorbent and analysed Porapak extract by GC-mass spectrometry, expecting to find many common blend constituents. Most volatiles were indeed shared between microbes, but a few others were unique to specific isolates (table 4). Interestingly, 2- and 3-methylbutyric acid and isobutyric acid were present in the odour blend of several microbes, implying that attraction of *I. scapularis* to butyric acid [28] was likely due to the shared resemblance of butyric acid with other microbial metabolites, thereby signalling the presence of a prospective host. Because many microbes also emit gases such as CO_2_ and NH_3_ [32], we tested for, and confirmed, emission of both gases by each of the four bioactive microbes (table 4).

Even though the Porapak Q HVE of A4 was complex (table 4), it was conceivable that essential microbe volatiles were still missing and would be obtainable only by using adsorbents such as Tenax and Carbosieve which possess adsorptive characteristics different than Porapak Q. Although Porapak Q, Tenax and Carbosieve are all porous polymers which trap volatiles in their porous structures [53], the compounds they trap vary in both molecular weight and size. Using Tenax and Carbosieve as adsorbents, and also by thermally desorbing volatiles from Tenax and Carbosieve in a headspace analyser, we indeed found compounds such as acetic acid and butanal which had remained below detection threshold in Porapak Q HVEs (electronic supplementary material, table S3).

To ascertain that essential volatiles of bioactive microbes were captured on Porapak Q/Tenax/Carbosieve, and that the extract was attractive to ticks, we used HVE of *Bacillus aryabhattai* A4 (HVE-A4) as a test case. Although HVE-A4 (4.8 PHE) on its own was not attractive to *I. pacificus* (figure 5, Exp. 43), it significantly enhanced the attraction of ticks to CO_2_ (figure 5, Exps. 44–45). In turn, CO_2_ significantly enhanced attraction of ticks to HVE-A4 (4.8 PHE; figure 5, Exp. 49), revealing a strong synergism between HVE-A4 and CO_2_ on tick attraction. As CO_2_ is emitted by A4 but not captured on any of the three adsorbents, we supplied CO_2_ from a separate source in the form of CO_2_-enriched medical air. Because the synergistic effect of HVE-A4 was dose-dependent, with 0.048 PHEs of HVE-A4 being ineffective (figure 5, Exp. 46), it follows that essential semiochemicals in HVE-A4 are attractive to ticks only over a relatively small concentration range. In the absence of volatile semiochemicals, the effect of CO_2_ on tick attraction was inconsistent (figure 5, Exps. 40, 42), likely due to (*i*) the contrasting modes of CO_2_ delivery, (*ii*) deviating CO_2_ release from the various test stimuli, and (*iii*) unquantifiable release of CO_2_ from an open source of ammonium bicarbonate in water and thus possibly an incorrect ratio of CO_2_ and NH_3_.

To test the effect of synthetic A4 volatiles on tick attraction, we prepared a synthetic blend (‘synthetic HVE-A4'; table 1) that contained 20 of the 27 A4 headspace volatiles that we had identified. Seven compounds had to be excluded from the synthetic blend because they were not obtainable in the time frame of our study. Like natural HVE-A4 (4.8 PHEs), synthetic HVE-A4 significantly enhanced the attractiveness of CO_2_ but only when tested at a 10-fold higher dose (48 PHEs) (figure 5, Exps. 50–51), suggesting that one or more of the seven volatiles not included in the synthetic blend play a role in tick attraction. This interpretation is supported by a study that tested questing behaviour by the cattle ticks *Boophilus microplus* and *Ixodes ricinus* in response to natural and synthetic bovine odour [18]. Here, progressively more complex blends of key components induced progressively stronger questing responses by ticks [18], but higher doses of single compounds could compensate for missing blend constituents.

The development of microbe- or host vertebrate-derived semiochemicals as trap lures for ticks, or as the attractants for ‘attract & kill' controls of ticks, seems feasible but is still challenging. Generally, host kairomones as tick attractants would be expected to have a broader appeal to ticks than pheromones, which typically are species-specific, although some tick species use the same pheromone [54]. And yet, kairomones differ between hosts, and many ticks seek specific hosts. For example, Asian blue ticks, *Rhipicephalus* (*Boophilus*) *microplus*, perform best on cattle hosts [55], whereas adult *I. pacificus* and *I. scapularis* seek deer hosts [21–23,56]. Even among tick species that seek the same host, different kairomones may trigger questing, host-seeking behaviour, or selection of micro-locations on hosts. For example, adult brown ear ticks, *Rhipicephalus appendiculatus,* and red-legged ticks, *R. evertsi*, prefer to feed inside ears and anal regions of bovids, respectively, and orient towards their respective feeding sites, with ear odour attracting *R. appendiculatus* but repelling *R. evertsi* and *vice versa* [3]. Resource partitioning of ticks was also described for two tick species that seek deer as their host. The winter tick, *Dermacentor albipictus*, was found almost exclusively on the head of deer, whereas *I. scapularis* was more evenly distributed on the body [4]. In our study, some microbes attracted *I. pacificus* whereas others attracted *I. scapularis*, and HVE-A4 in combination with CO_2_ attracted *I. pacificus* but not *I. scapularis* (figure 5; Exps. 44–45, 47–48). Also, there is limited knowledge as to whether host location and recognition by ticks involves the detection of both host attractants and non-host repellents. Brown dog ticks, *Rhipicephalus sanguineus,* discern between tick-susceptible dogs (English cocker spaniels) and tick-resistant dogs (beagles) [57]. In a comparative odour profile study, tick-resistant dogs (beagles, miniature pinschers) produced more benzaldehyde, 2-hexanone and 1,2,4-trimethylbenzene than tick-susceptible cocker spaniels, and miniature pinschers produced more 6-methyl-5-hepten-2-one than the other two breeds of dogs, with 6-methyl-5-hepten-2-one by itself being deterrent to ticks [58]. These results suggest that host selection by ticks is mediated, in part, by the relative amount of non-host deterrents present in the odour profile of potential hosts. Similarly, preference by the blood-feeding tsetse flies *Glossina morsitans* and *G. pallidipes* for buffalo and ox hosts over the waterbuck non-host is mediated by compounds (nearly) absent from the two preferred hosts [59].

In conclusion, our data support the hypothesis that host-foraging Western black-legged ticks respond to microbes dwelling in sebaceous gland secretions of white-tailed deer, the ticks' preferred host. Of the 31 microbial isolates, some were attractive to ticks, whereas others were deterrent or indifferent. Deterrent microbes may play a role in guiding ticks away from micro-locations of deer skin that are less suitable or preferred for obtaining a blood meal. Attraction of ticks to *Bacillus aryabhattai* (isolate A4), the microbial isolate most attractive to ticks, was mediated by a—heretofore unknown—synergistic interaction between CO_2_ and volatile odorants emitted by *B. aryabhattai* (A4). The aim of future tick research should be directed to the development of lures that disseminate both CO_2_ and a least complex blend of odorants (void of non-host deterrents) that are attractive to diverse tick taxa.

## Data Availability

Additional data are provided in the electronic supplementary material [60].

## References

[1] Carr A, Roe M. 2016 Acarine attractants: chemoreception, bioassay, chemistry and control. Pestic. Biochem. Physiol. **131**, 60-79. (10.1016/j.pestbp.2015.12.009) 27265828 PMC4900186

[2] Sonenshine D, Roe M. 2014 Biology of ticks. New York, NY: Oxford University Press.

[3] Wanzala W, Sika N, Gule S, Hassanali A. 2004 Attractive and repellent host odours guide ticks to their respective feeding sites. Chemeocology **14**, 229-232. (10.1007/s00049-004-0280-6)

[4] Baer-Lehmen M, Light T, Fuller N, Barry-Landis K, Kindlin C, Stewart Jr R. 2012 Evidence for competition between *Ixodes scapularis* and *Dermacentor albipictus* feeding concurrently on white-tailed deer. Exp. Appl. Acarol. **58**, 301-314. (10.1007/s10493-012-9574-5) 22644381

[5] Garcia R. 1962 Carbon dioxide as an attractant for certain ticks (Acarina: Argasidae and Ixodidae). Ann. Entomol. Soc. Am. **55**, 605-606. (10.1093/aesa/55.5.605)

[6] Carroll F. 2002 Responses of host-seeking nymphs and adults of the ticks *Ixodes scapularis* and *Amblyomma americanum* (Acari: Ixodidae) to canine, avian and deer-produced substances. Proc. Entomol. Soc. Wash. **104**, 73-78.

[7] Carroll JF. 2001 Interdigital gland substances of white-tailed deer and the response of host-seeking ticks (Acari: *Ixodidae*). Med. Entomol. **38**, 114-117. (10.1603/0022-2585-38.1.114) 11268681

[8] Carr A, Salgado V. 2019 Ticks home in on body heat: a new understanding of Haller's organ and repellent action. PLoS ONE **14**, e0221659. (10.1371/journal.pone.0221659) 31442282 PMC6707551

[9] Mitchell R, Zhu J, Carr A, Dhammi A, Cave G, Sonenshine D, Roe M. 2017 Infrared light detection by the Haller's organ of adult American dog ticks, *Dermacentor variabilis* (Ixodida: Ixodidae). Ticks Tick-borne Dis. **8**, 764-771. (10.1016/j.ttbdis.2017.06.001) 28647127 PMC5588665

[10] Suss J, Klaus C, Gerstengarbe F, Werner P. 2008 What makes ticks tick? Climate change, ticks, and tick-borne diseases. J. Travel Med. **1**, 39-45. (10.1111/j.1708-8305.2007.00176.x) 18217868

[11] Carr A, Mitchell R, Dhammi A, Bissinger B, Sonenshine D, Roe M. 2017 Tick Haller's organ, a new paradigm for arthropod olfaction: how ticks differ from insects. Int. J. Mol. Sci. **18**, 1563. (10.3390/ijms18071563) 28718821 PMC5536051

[12] Sonenshine D. 2004 Pheromone and other semiochemicals of ticks and their use in tick control. Parasitology **7**, 405-425. (10.1017/S003118200400486X) 15938521

[13] Carroll JF. 1999 Responses of three species of adult ticks (*Acari: Ixodidae*) to chemicals in the coats of principal and minor hosts. Med. Entomol. **36**, 238-241. (10.1093/jmedent/36.3.238) 10337091

[14] Carroll JF. 1998 Kairomonal activity of white-tailed deer metatarsal gland substances: A more sensitive behavioral bioassay using *Ixodes scapularis* (Acari: Ixodidae). J. Med. Entomol. **35**, 90-93. (10.1093/jmedent/35.1.90) 9542351

[15] Smallegange RC, Verhulst NO, Takken W. 2011 Sweaty skin: an invitation to bite? Trends Parasitol. **27**, 143-148. (10.1016/j.pt.2010.12.009) 21256083

[16] Kenneth KK. 2015 Culturing, characterization and identification of candidate microorganisms in cattle ear, responsible for producing volatile constituents attractive to the brown ear tick. MSc. thesis, School of Pure and Applied Sciences, Kenyatta University, Kenya.

[17] Steullet P, Guerin P. 1992 Perception of breath components by the tropical bont tick, *Amblyomma variegatum* Fabricius (Ixodidae). I. CO_2_-excited and CO_2_-inhibited receptors. Comp. Physiol. **170**, 665-676. (10.1007/BF00198976) 1331433

[18] Osterkamp J, Wahl U, Schmalfuss J, Hass W. 1999 Host-odour recognition in two tick species is coded in a blend of vertebrate volatiles. Comp. Physiol. **185**, 59-67. (10.1007/s003590050366) 10450611

[19] Leonovich S. 2004 Phenol and lactone receptors in the distal sensilla of the Haller's organ in *Ixodes ricinus* ticks and their possible role in host perception. Exp. App. Acarol. **32**, 89-102. (10.1023/b:appa.0000018200.24760.78) 15139275

[20] Eisen R, Eisen J, Beard C. 2017 Country-scale distribution of *Ixodes scapularis* and *Ixodes pacificus* (*Acari: Ixodidae*) in the continental United States. Med. Entomol. **53**, 349-386. (10.1093/jme/tjv237) PMC4844559 26783367

[21] Aucott J, Luft B. 2017 46 – lyme disease. J. Infect. Dis. **4**, 405-414. (10.1016/B978-0-7020-6285-8.00046-0)

[22] Sudhindra P. 2018 Chapter 10 – tick-borne infections of the central nervous system. Clin. Microbiol. Diag. **3**, 173-195. (10.1016/B978-0-12-813806-9.00010-X)

[23] McVicar M, Rivera I, Reyes JB, Gulia-Nuss M. 2022 Ecology of *Ixodes pacificus* ticks and associated pathogens in the Western United States. Pathogens **11**, 89. (10.3390/pathogens11010089) 35056037 PMC8780575

[24] Tiffin HS, Skvarla MJ, Machtinger ET. 2021 Tick abundance and life-stage segregation on the American black bear (*Ursus americanus*). Int. J. Parasitol.: Parasites Wildl. **11**, 208-216. (10.1016/j.ijppaw.2021.10.004) PMC8523825 34703760

[25] Kilpatrick H, LaBonte A, Stafford K. 2014 The relationship between deer density, tick abundance, and human cases of Lyme disease in a residential community. Med. Entomol. **1**, 777-784. (10.1603/me13232) 25118409

[26] Centers for Disease Control and Prevention. 2021 Lyme disease. See https://www.cdc.gov/lyme/stats/humancases.html.

[27] Field & Stream. 2009 Understanding seven deer glands. See https://www.fieldandstream.com/articles/hunting/2009/11/how-whitetail-glands-work/.

[28] Faraone N, Light M, Scott C, MacPherson S, Hillier K. 2020 Chemosensory and behavioral responses of *Ixodes scapularis* to natural products: role of chemosensory organs in volatile detection. Insects **8**, 1-13. (10.3390/insects11080502) PMC7469143 32759735

[29] Sheehan J. 2022 Avoidance of gas blowing. Encycl. Dairy Sci. **3**, 15-21. (10.1016/B978-0-12-818766-1.00310-X)

[30] Ryu C, Farag M, Hu C, Kloepper J. 2003 Bacterial volatiles promote growth in *Arabidopsis*. Proc. Natl Acad. Sci. USA **100**, 4927-4932. (10.1073/pnas.0730845100) 12684534 PMC153657

[31] Biotechnology and Biology of Trichoderma. 2014 Secondary Metabolite. See https://www.sciencedirect.com/topics/agricultural-and-biological-sciences/secondary-metabolite.

[32] Audrain B, Farag MA, Ryu C, Ghigo J. 2015 Role of bacterial volatile compounds in bacterial biology. Fed. Eur. Microbiol. Soc. Microbiol. Rev. **39**, 222-233. (10.1093/femsre/fuu013) 25725014

[33] Gao H, Li P, Xu X, Zeng Q, Guan W. 2018 Research on volatile organic compounds from *Bacillus subtilis* CF-3: biocontrol effects on fruit fungal pathogens and dynamic changes during fermentation. Front. Microbiol. **9**, 456. (10.3389/fmicb.2018.00456) 29593695 PMC5861295

[34] Netzker T, Shepherdson E, Zambri M, Elliot M. 2020 Bacteria volatile compounds: function in communication, cooperation, and competition. Annu. Rev. Microbiol. **74**, 409-430. (10.1146/annurev-micro-011320-015542) 32667838

[35] Yoder J, Stevens B. 2000 Attraction of immature stages of the American dog tick (*Dermacentor variabilis*) to 2,6-dichlorophenol. Exp. Appl. Acarol. **24**, 159-164. (10.1023/A:1006419203251) 11108396

[36] Carr A, Roe M, Arellano C, Sonenshine D, Schal C, Apperson C. 2013 Responses of *Amblyomma americanum* and *Dermacentor variabilis* to odorants that attract haematophagous insects. Med. Vet. Entomol. **27**, 86-95. (10.1111/j.1365-2915.2012.01024.x) 22681499

[37] Poldy J. 2020 Volatiles cues influence host-choice in arthropod pest. Animals **10**, 1984. (10.3390/ani10111984) 33126768 PMC7692281

[38] Van Breugel F, Riffell J, Fairhall A, Dickinson M. 2015 Mosquitoes use vision to associate odor plumes with thermal targets. Curr. Biol. **25**, 2123-2129. (10.1016/j.cub.2015.06.046) 26190071 PMC4546539

[39] Wooding M, Naude Y, Rohwer E, Bouwer M. 2020 Controlling mosquitoes with semiochemicals: a review. Parasites Vectors **13**, 80. (10.1186/s13071-020-3960-3) 32066499 PMC7027039

[40] Alberto AS, Rusch CD, Zhan Y, Straw AD, Montell C, Riffell JA. 2022 The olfactory gating of visual preferences to human skin and visible spectra in mosquitoes. Nat. Commun. **13**, 555. (10.1038/s41467-022-28195-x) 35121739 PMC8816903

[41] Johnson JS et al. 2019 Evaluation of 16S rRNA gene sequencing for species and strain-level microbiome analysis. Nat. Commun. **10**, 5029. (10.1038/s41467-019-13036-1) 31695033 PMC6834636

[42] Takács S, Gries G. 2001 Communication ecology of webbing clothes moth: attractiveness and characterization of male-produced sonic aggregation signal(s). Can. Entomol. **133**, 725-727. (10.1046/j.1439-0418.2003.00724.x)

[43] Siljander E, Gries R, Khaskin G, Gries G. 2008 Identification of the airborne aggregation pheromone of the common bed bug, *Cimex lectularius*. J. Chem. Ecol. **34**, 708-718. (10.1007/s10886-008-9446-y) 18470566

[44] Van Den Dool H, Kratz PD. 1963 A generalization of the retention index system including linear temperature programmed gas–liquid partition chromatography. J. Chromatogr. **2**, 463-471. (10.1016/S0021-9673(01)80947-X) 14062605

[45] Masuda H, Yoshida M, Shibamoto T. 1981 Synthesis of new pyrazines for flavor use. J. Agric. Food Chem **29**, 944-947. (10.1021/jf00107a014)

[46] Mihara S, Masuda H. 1990 Structure elucidation of the product prepared from the reaction of 3-methyl-5,6-dihydro-2(1H)-pyrazinone and ketones or aldehydes. J. Agric. Food Chem. **38**, 1032-1034. (10.1021/jf00094a025)

[47] RStudio Team. 2021 RStudio: integrated development environment for R. Boston, MA: RStudio, PBC. See http://www.rstudio.com/.

[48] Verhulst N, Weldegeris B, Menger D, Takken W. 2016 Attractiveness of volatiles from different body parts to the malaria mosquito *Anopheles coluzzii* is affected by deodorant compounds. Sci. Rep. **6**, 27141. (10.1038/srep27141) 27251017 PMC4890431

[49] Martinez J, Showering A, Oke C, Jones R, Logan J. 2020 Differential attraction in mosquito-human interactions and implications for disease control. Phil. Trans. R. Soc. B **376**, 20190811. (10.1098/rstb.2019.0811) 33357061 PMC7776937

[50] Ellwanger J, Cardoso J, Chies J. 2021 Variability in human attractiveness to mosquitoes. Curr. Res. Parasitol. Vector-Borne Dis. **1**, 100058. (10.1016/j.crpvbd.2021.100058) 35284885 PMC8906108

[51] Alexy K, Gassett J, Osborn DA, Miller K. 2003 Bacterial fauna of the tarsal tufts of white-tailed deer (*Odocoileus virginianus*). Am. Midl. Nat. **149**, 237-240. (10.1674/0003-0031(2003)149[0237:BFOTTT]2.0.CO;2)

[52] Kai M. 2020 Diversity and distribution of volatile secondary metabolites throughout *Bacillus subtilis* isolates. Front. Microbiol. **11**, 559. (10.3389/fmicb.2020.00559) 32322244 PMC7156558

[53] Cao X, Hewitt N. 1993 Evaluation of Tenax-GR adsorbent for the passive sampling of volatile organic compounds at low concentrations. Atmos. Environ. **27**, 1865-1872. (10.1016/0960-1686(93)90291-6)

[54] Petney N, Andrews H. 1982 The influence of similar aggregation pheromones on the microhabitat choice of two parapatric species of reptile tick (Acari: Ixodidae). Oecologia **55**, 364-368. (10.1007/BF0037692) 28309977

[55] Ma M, Chen Z, Liu A, Ren Q, Liu J, Li Y, Yin H, Guan G, Luo J. 2016 Biological parameters of *Rhipicephalus* (*Boophilus*) *microplus* (Acari: Ixodidae) fed on rabbits, sheep, and cattle. Korean J. Parasitol. **54**, 301-305. (10.3347/kjp.2016.54.3.301) 27417084 PMC4977778

[56] Watson TG, Anderson R. 1976 *Ixodes scapularis* Say on white-tailed deer (*Odocoileus virginianus*) from long point, Ontario. J. Wildl. Dis. **12**, 66-71. (10.7589/0090-3558-12.1.66) 1255915

[57] Louly CCB, Soares SF, da Nóbrega Silveira D. 2010 Differences in the behavior of *Rhipicephalus sanguineus* tested against resistant and susceptible dogs. Exp. Appl. Acarol. **51**, 353-362. (10.1007/s10493-009-9334-3) 20091335

[58] Zeringóta V, Pereira-Junior RA, Sarria ALF, Henrique ACC, Birkett MA, Borges LMF. 2021 Identification of a non-host semiochemical from miniature pinscher, *Canis lupus familiaris*, that repels *Rhipicephalus sanguineus sensu lato* (Acari: Ixodidae). Ticks Tick-borne Dis. **12**, 101582. (10.1016/j.ttbdis.2020.101582) 33038704

[59] Gikonyo N, Hassanali A, Njagi P, Gitu P, Midiwo J. 2002 Odor composition of preferred (buffalo and ox) and nonpreferred (waterbuck) hosts of some Savanna tsetse flies. Chem. Ecol. **28**, 969-981. (10.1023/A:1015205716921) 12049234

[60] Long J, Maskell K, Gries R, Nayani S, Gooding C, Gries G. 2023 Synergistic attraction of western black-legged ticks, *Ixodes pacificus,* to CO_2_ and odorant emissions from deer-associated microbes. Figshare. (10.6084/m9.figshare.c.6631205) PMC10189596 37206969

